# On the Conservation of the Slow Conformational Dynamics within the Amino Acid Kinase Family: NAGK the Paradigm

**DOI:** 10.1371/journal.pcbi.1000738

**Published:** 2010-04-08

**Authors:** Enrique Marcos, Ramon Crehuet, Ivet Bahar

**Affiliations:** 1 Department of Biological Chemistry and Molecular Modelling, IQAC-CSIC, Barcelona, Spain; 2 Department of Computational Biology, School of Medicine, University of Pittsburgh, Pittsburgh, Pennsylvania, United States of America; Stanford University, United States of America

## Abstract

N-Acetyl-*L*-Glutamate Kinase (NAGK) is the structural paradigm for examining the catalytic mechanisms and dynamics of amino acid kinase family members. Given that the slow conformational dynamics of the NAGK (at the microseconds time scale or slower) may be rate-limiting, it is of importance to assess the mechanisms of the most cooperative modes of motion intrinsically accessible to this enzyme. Here, we present the results from normal mode analysis using an elastic network model representation, which shows that the conformational mechanisms for substrate binding by NAGK strongly correlate with the intrinsic dynamics of the enzyme in the unbound form. We further analyzed the potential mechanisms of allosteric signalling within NAGK using a Markov model for network communication. Comparative analysis of the dynamics of family members strongly suggests that the low-frequency modes of motion and the associated intramolecular couplings that establish signal transduction are highly conserved among family members, in support of the paradigm sequence→structure→dynamics→function.

## Author Summary

During the last 20 years both the experimental and computational communities have provided strong evidence that proteins cannot be regarded as static entities, but as intrinsically flexible molecules that exploit their fluctuation dynamics for catalytic and ligand-binding events, as well as for allosteric regulation. This intrinsic dynamics is encoded in the protein structure and, therefore, those proteins with similar folding should share dynamic features essential to their biological function. In this work, we have applied an Elastic Network Model to predict the large-amplitude dynamics of different enzymes belonging to the same protein family (Amino Acid Kinase family). Subsequent comparison of the dynamics of these proteins reveals that this protein family follows the same dynamic pattern. The present results are strongly supported by experimental data and provide new insights into the performance of biological function by these enzymes. The investigation presented here provides us with a useful framework to identify dynamic fingerprints among proteins with structural similarities.

## Introduction

Many recent studies, both experimental and computational, point to the inherent ability of proteins to undergo, under native state conditions, large-amplitude conformational changes that are usually linked to their biological function. Proteins have access, via such equilibrium fluctuations, to an ensemble of conformers encoded by their 3-dimensional (3D) structure; and ligand binding essentially shifts the population of these pre-existing conformers in favour of the ligand-bound form [1]–[4]. With the accessibility of multiple structures resolved for a given protein in different forms, it is now possible to identify the principal changes in structure assumed by a given protein upon binding different ligands, which are observed to conform to those intrinsically accessible to the protein prior to ligand binding [5]–[7]. The observations suggest the dominance of proteins' intrinsic dynamics in defining the modes of interactions with the ligands. This is in contrast to the induced-fit model [8] where the ligand ‘induces’ the change in conformation. Instead, the Monod-Wyman-Changeux (MWC) [9] model of allostery where a selection from amongst those conformers already accessible is triggered upon ligand binding.

Yet, the choice between intrinsic *vs* induced dynamics, and the correlations between dynamics and function, are still to be established, and presumably depend on the particular systems of study [10]. NMR relaxation experiments provide evidence, for example, for the existence of correlations between the time scales of large-amplitude conformational motions and catalytic turnover [11],[12]; and collective motions in the low frequency regime appear to be potentially limiting reaction rates. On the other hand, other studies point to the different time scales and events that control catalysis and binding events [13],[14]. Furthermore, while the intrinsic dynamics in the unbound form is observed to be the dominant mechanism that facilitates protein-protein or protein-ligand complexation, the ligand may also promote structural rearrangements on a local scale at the binding site [2],[15],[16]. Given that proteins' collective dynamics, and thereby potential functional motions, are encoded by the structure, proteins grouped in families on the basis of their fold similarities would be expected to share relevant dynamical features [17]–[21]. It is of paramount importance, in this respect, to have a clear understanding of collective motions and their relationship to binding or catalytic activities, if any, toward gaining deeper insights into functional mechanisms shared by members of protein families.

Protein dynamics can be explored by means of all-atom force fields and simulations, or by coarse-grained (CG) models and methods. All-atom simulations such as Molecular Dynamics (MD) describe the conformational fluctuations of the system over a broad range of timescales. Except for small proteins, the main limitation of MD is that the timescales computationally attainable (below hundreds of nanoseconds) do not allow for accurate sampling of slow and large-amplitude motions (low-frequency modes) that are usually of biological interest. CG approaches, on the other hand, lack atomic details but provide insights into global movements. Among them, Elastic Network Models (ENMs) have found wide use in conjunction with normal mode analyses (NMAs) in the last decade [22]. ENMs describe the protein as a network, the nodes of which are usually identified by the spatial positions of C^α^-atoms. Elastic springs of uniform force constant connect the nodes in the simplest (most broadly used) ENM, referred to as the anisotropic network model (ANM) [23]–[25]. Despite the oversimplified description of the protein conveyed by the ENMs, a surge of studies have shown that the predicted low-frequency modes describe well experimentally observed conformational changes and provide insights into potential mechanisms of function and allostery [5]–[7], [24]–[27], in accord with NMAs performed [28],[29] with more detailed models and force fields. Additionally, recent studies by Orozco and co-workers [30], and Liu et al [31] point to the similarities of the conformational space described by the low-frequency modes obtained from MD and that from CG NMA, provided that MD runs are long enough to accurately sample the collective motions.

The present study focuses on the amino acid kinase (AAK) family. This family comprises the following enzymes on the basis of sequence identity and structural similarities: N-acetyl-L-glutamate (NAG) kinase (NAGK), carbamate kinase (CK), glutamate-5-kinase (G5K), UMP kinase (UMPK), aspartokinase (AK) and the fosfomycin resistance kinase (FomA). Rubio and co-workers [32] have exhaustively studied this family and proposed that the shared fold among the members is likely to give rise to a similar mechanism of substrate binding and catalysis. NAGK is the most widely studied member of this family taking into account the large amount of structural information gathered [32],[33]. This enzyme indeed serves as a structural paradigm for the AAK family, such that studying its structure-encoded dynamics can shed light on the mechanisms shared by family members to perform their function [32].

**Figure 1:**
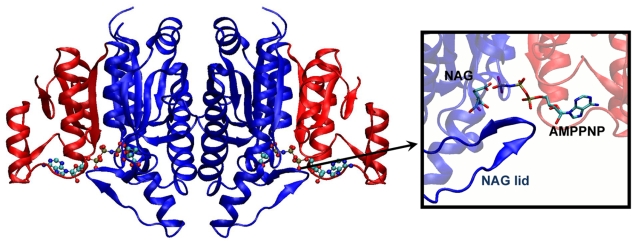
Structure of the closed form of NAGK. The NAGK dimer is complexed with the ATP analogue AMPPNP and the amino acid NAG (PDB code 1GS5) [32]. The substrates ATP and NAG (ball and sticks) bind to the C- (red) and N-domains (blue) respectively. N-terminal domains form the interface between the two monomers. Inset shows a closer view of the NAG lid and both ligands.

NAGK catalyzes the phosphorylation of NAG, which is the controlling step in arginine biosynthesis. The hallmark of this biosynthetic route in bacteria is that it proceeds through N-acetylated intermediates, as opposed to mammals that produce non-acetylated intermediates. Consequently, NAGK activity may be selectively inhibited and, taking into account that it is the controlling enzyme of arginine biosynthesis, it is a potential target for antibacterial drugs. In many organisms, NAGK phosphorylation is the controlling step in arginine biosynthesis. In these cases, NAGK is feedback inhibited by the end product arginine, and recent studies shed light on this mechanism of inhibition [34],[35]. NAGK from *Escherichia Coli* (*Ec*NAGK), on the other hand, is arginine-insensitive. Its mechanism of phosphoryl transfer has been the most thoroughly characterized among the enzymes that catalyze the synthesis of acylphosphates (EC group 2.7.2). In particular, crystallographic studies by Rubio and coworkers [32],[33] have provided insights into its mechanisms of binding and catalysis. *Ec*NAGK is a homodimer of 258 residues, each monomer being folded into an αβα sandwich (Figure 1). The N-domain of each subunit/monomer makes intersubunit contacts and hosts the NAG binding site (NAG lid), whereas the C-domain binds the ATP. The phosphoryl transfer reaction takes place at the interface between the two domains within each subunit. Kinetic studies show no evidence of cooperativity between subunits [36], suggesting that the dimeric structure provides thermodynamic stability, only, to the monomeric fold that has been evolutionary selected to perform the catalytic function.

The diverse crystallographic structures solved for the bound state of this enzyme indicate two types of functional motions [33]: (1) X-ray structures of *Ec*NAGK complexed with either ADP or with the inert ATP analogue AMPPNP (PDB codes 1GS5, 1OH9, 1OHA and 1OHB) have a too narrow active site to let the substrates bind directly; whereas the unbound structure (PDB code 2WXB; kindly provided by the authors prior to release) has a more open active site. This suggests that the enzyme undergoes a conformational closure that is likely to be triggered upon nucleotide binding, since all these complexes display a closed structure whether NAG is bound or not. (2) The ternary complex with ADP and NAG displays the ability to exchange NAG with a sulphate ion in solution without opening the active site. The NAG lid therefore must be able to open and close independently of other structural elements.

The aim of the present study is two-fold. Firstly, given the interest in acquiring deeper knowledge on the enzymatic mechanism of *Ec*NAGK and the potential role of slow dynamics in the pre-disposition of the enzymatic function, we analyze here the low-frequency modes of motion of *Ec*NAGK. Secondly, using *Ec*NAGK as the paradigm of AAK family, we assess to what extent the slow modes of motion are shared by other members of the AAK family.

## Results/Discussion

### Plan

The results are organized as follows. First, results from GNM analysis are presented, which give insights into the functional significance of residue fluctuations and underlying sizes of motions in the most readily accessible (i.e. softest) collective modes. Second, ANM modes are described to analyze the directionality/mechanism of these modes. Note that GNM does not provide information on 3N-dimensional structural changes, but on N-dimensional properties such as the mean-square fluctuations (MSFs) of residues, their cross-correlations, or movements along normal mode axes, hence the use of the ANM for exploring and visualizing the 3D motions (see *Methods*). Third, communication properties of the *Ec*NAGK enzyme are assessed based on graph theoretical examination of shortest paths between network nodes representative of the enzyme. Finally, a comparative analysis of the ANM dynamics of different members of the AAK family is made. GNM and ANM modes of *Ec*NAGK are computed for the open form in general, except for the analysis of the intrinsic dynamics of the closed form; and ligands are not included in the calculations. The predicted motions therefore reflect the intrinsic dynamics of the enzyme in the absence of bound ligands.

### Mobility profiles for the EcNAGK open form

**Figure 2:**
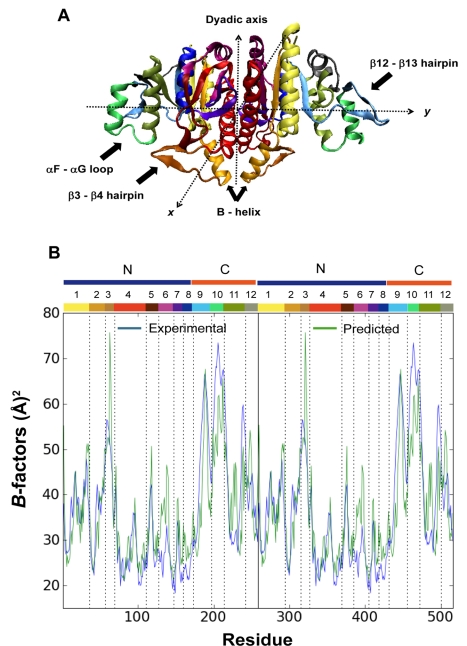
Structure and fluctuation dynamics of the open conformation of NAGK. (A) Color-coded ribbon diagram of NAGK where regions involved in substrate binding are indicated by arrows. All secondary structure elements are highlighted with different colors. (B) Comparison of experimentally observed (blue) and computationally predicted (green) *B*-factors. The theoretically predicted *B*-factors are rescaled based on the experimentally observed *B*-factors averaged over all residues. Experimental data refer to the PDB structure 2WXB (to be published). The range of residues of N and C domains is highlighted. The different parts of the protein have been numbered as follows: (1) β1+αA; (2) β2+αB; (3) β3–β4, the NAG lid; (4) αC+β5; (5) β6–β7; (6) αD+β8; (7) β9–β10;(8) αE; (9) β11+β12–β13+β14; (10) αF+αF–αG; (11) αG+β15; (12) αH+β16. The color code of the numbered parts of the protein is the same in both subunits, and indicated along the upper abscissa.

Figure 2 displays the results from the GNM analysis of the equilibrium dynamics of *Ec*NAGK. Panel (B) compares the MSFs of residues, <(Δ*R_i_*)^2^>, predicted by the GNM with those indicated by X-ray crystallographic *B*-factors *B_i_* = 8π^2^/3 <(Δ*R_i_*)^2^>. For clarity, the different structural elements are numbered and color-coded along the upper abscissa bar in accord with the colors in panel (A). Results for chains A and B are identical as a result of the dyadic axis of symmetry at the intersubunit surface. Calculations and experimental data refer to the open form of *Ec*NAGK. The high correlation coefficient (r = 0.75) between the experimental and theoretical curves in Figure 2 is remarkable in view of the simplicity of the GNM, but it is also worth noting that in the case of the closed conformation the correlation coefficient drops to r = 0.61. Indeed, ENMs tend to provide a better description of the dynamics of open forms [24].

The mobility profile in Figure 2B permits us to identify the most mobile and rigid regions of the protein from the maxima and minima, respectively. Mainly two dynamical features are distinguished. First, the β3–β4 hairpin, which corresponds to the NAG-binding site lid, is the most mobile part of the N-domain (region 3). Second, the β12–β13 hairpin and αF and αG helices (regions 9 and 10) emerge as the most mobile parts of the C-domain; notably, these structural elements are involved in ATP binding. It is remarkable that the topology of the structure provides flexibility near the two binding sites, which may be a functional requirement to accommodate ligand binding. Rigid/constrained elements, on the other hand, include the αC helices making intersubunit contacts, along with the strands β8 and β10 in the N-domain core. Moreover, the N-termini of αB and αE helices (regions 2 and 8), which point toward the γ-phosphate in the closed form, also show reduced mobility. The lack of mobility in these NAGK sequence motifs [32] is presumably a dynamic requirement to optimally perform their functional role in orienting their dipoles to withdraw negative charge from the transferring phosphate group. These results are consistent with those inferred by Rubio and co-workers from their crystallographic studies [32],[33],

### Global modes point to intrinsic mechanisms for opening/closing ligand binding sites

The decomposition of the global dynamics into a set of GNM modes permits us to identify the different kinds of motions allowed by the structure as well as the couplings between different parts of the protein. Moreover, minima in the low-frequency mode-profiles reveal mechanically important residues. When residues surrounding a given site move in opposite directions, the latter site serves as a hinge. Hinge sites at low-frequency modes, also called soft modes, usually serve as key mechanical site at the interface between domains subject to concerted movements [37].

**Figure 3:**
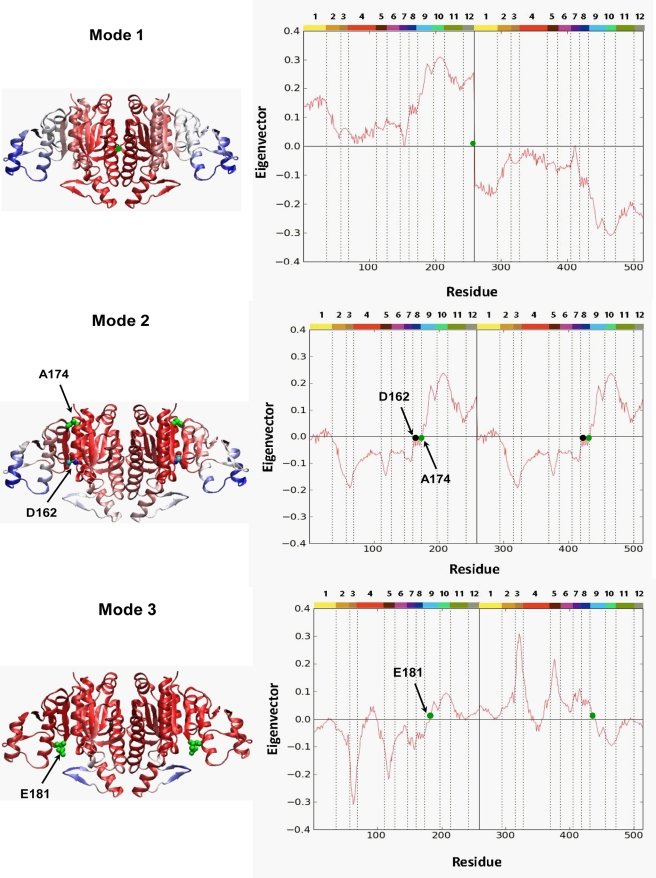
Mobilities of residues. Results are presented for the softest three GNM modes. On the left, the ribbon diagrams of NAGK, color-coded according to the mobilities of residues in the respective modes are displayed. The most mobile residues are colored blue, and the most rigid ones, red. The green dots on the diagrams indicate the position of the hinge sites. Note that in mode 2 the hinge sites closely neighbour the residue D162, which is a key catalytic residue.

Figure 3 shows the mobility of different parts of the protein in the first three softest modes. The diagrams are color coded from red (most rigid) to blue (most mobile), in accord with the size of motions undergone by the residues along these examined modes' axes (shown on the right panels). All three modes appear to induce motions symmetrically distributed about the inter-subunit interface. In the 1^st^ mode, the mobility increases with distance away from the dyadic axis, such that the C-domain, and in particular the β12–β13 hairpin and αF helix, undergo the largest movements.

The 2^nd^ slowest mode involves movements of the C- and N-domains with respect to each other within each monomer. A hinge site at residue A174 is observed, where previous crystallographic studies had exactly set the boundary between the C- and N-domains [32]. This hinge presumably enables the opening/closure of the active site in each subunit. A mutant on residue D162 [36], which is close to this hinge site, disrupted function and thus confirms this hinge as a key element in the functionality of the enzyme.

On the other hand, the 3^rd^ mode involves mainly the β3–β4 hairpin, i.e., the NAG lid, and suggests an intrinsic ability at this region to move independently with respect to the ATP site (note that all modes are orthonormal and independent). Such local flexibility is consistent with an ability of the NAG lid to open and close the NAG binding site, in support of the hypothesis inferred from crystallographic studies [33]. The anticorrelated motion of the C-domain, due to a hinge at residue E181, is minimal but, as in mode 2 and together with the movement of NAG lid, might lead to the opening/closure of the active site. Interfacial residues making intersubunit contacts exhibit low mobilities, suggesting that the tightly packed hydrophobic contacts are essential to the thermodynamic stability of the dimeric protein.

### How do global modes correlate with experimentally observed change in structure?

With the aim of gaining insights into the directionality of these modes, one can map GNM modes into ANM modes by comparing the mean-square fluctuations. A one-to-one correspondence would not be expected, due to differences in the number of modes as well as underlying potentials of the two ENMs. We found that the first GNM mode correlates with the 1^st^ and 3^rd^ ANM modes; the 2^nd^ with the 1^st^, 2^nd^ and 4^th^ ANM modes; and the 3^rd^ is the counterpart of the 5^th^ ANM mode.

**Figure 4:**
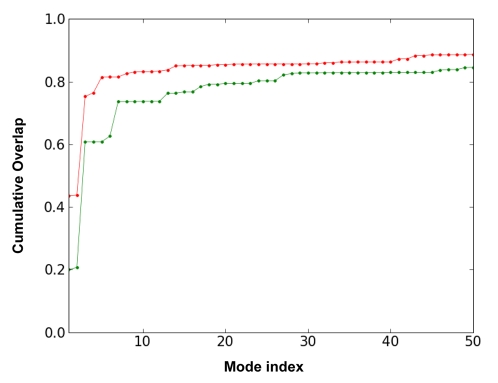
Comparison with experimental conformational changes. Cumulative overlaps CO(*m*) between ANM modes and the experimentally observed deformation between the open and closed forms of NAGK are plotted for subsets of m modes, in the range 1≤m≤50 (see equation (7) in *Methods*). We note that the first 3 ANM modes (among 3*N*-6 = 1542 modes) accessible to the open form (red) yield an overlap of 0.75 with the experimentally observed reconfiguration from open to closed state of the enzyme. In the case of the closed form, the first 3 modes yield an overlap of 0.61. In either case, a small subset of modes intrinsically accessible to the structure attain a cumulative overlap of >0.80, pointing to the pre-disposition of the structure to undergo its functional changes in conformation between the open and closed forms.

The directionality provided by the ANM approach helps us ascertain how well the slowest ANM modes describe the conformational difference observed between the open and closed structures resolved by X-ray crystallography. To this aim, the ANM modes are projected into the deformation vector **Δ** ***r*** obtained from the open and closed conformations. Figure 4 displays the cumulative overlap (see equation (7)) between **Δ** ***r*** and the ANM modes for both passages (from closed to open, and *vice versa*). It is worth emphasizing that the first 10 ANM modes of the open and closed forms are able to describe 84% and 76% of the observed conformational change, respectively. On the other hand, the open form requires a smaller set of modes to describe the deformation vector to a given extent. This is in agreement with the fact that the dynamics of open conformations are usually better described with ENM as also noted above. Modes 1, 3 and 5 are the main contributors to the cumulative overlap and thus the most relevant modes to describe the global dynamics of NAGK (see Figure S1).

### Changes induced near active sites by global modes

As mentioned above three modes play a dominant role in enabling the functional changes in NAGK. Modes 1 and 3 drive a symmetrical opening and closing of the active site. In both modes, the most mobile region is the C-domain, which binds ATP, while the N-domain is practically rigid. Mode 5 ensures the opening/closing of the NAG-binding site by the β3–β4 hairpin that serves as a lid.

**Figure 5:**
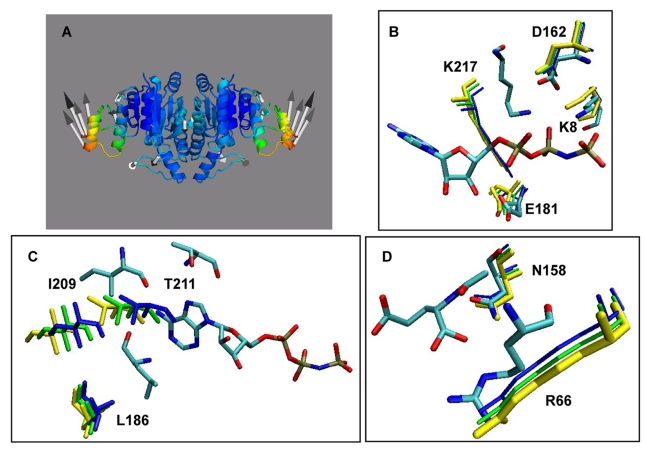
Movement along the slowest ANM mode. Motion of active site residues between open and closed conformers along the 1^st^ ANM mode accessible to the open form. The position of these residues in different conformations is shown: open conformation (yellow), intermediate positions (green and blue) and closed conformation (atom-colored). (A) Color-coded ribbon diagram for motions along the 1^st^ mode (generated with the ANM web server [25] and Pymol [64]). (B) Movement of catalytic residues with respect to the ATP analogue. (C) Movement of ATP binding residues with respect to the nucleotide. (D) Movement of NAG binding residues with respect to NAG.

It is of the utmost importance to examine how active site residues move in these modes. Do they possess an intrinsic ability to adopt the conformation of the bound state, or does ligand binding trigger the conformational change? To explore this issue, we generated a series of conformations driven by these modes. Figure 5 illustrates the results for mode 1. This mode entails an anticorrelated movement of the two subunits as shown in panel (A). The following features are distinguished by a closer examination of functional sites. The catalytic residues K8, K217 and D162, and those in the vicinity, such as N158, exhibit minimal changes in their coordinates as seen in panel (B). On the other hand, a number of hydrophobic residues near the ATP binding site move concertedly in a direction required for coordinating ATP, as the subunit reconfigures from the open to the closed form (see panel (C)). This mode accessible in the absence of ATP binding thus facilitates the suitable re-positioning of these residues upon ATP binding. NAG-binding residues undergo minimal change during this global motion (panel (D)). The movement of residues in mode 3 complement those in the 1^st^ mode to reach the bound conformation from the open form (Figure S2).

K8 and D162 are key catalytic residues on the basis of structural [32],[33] and mutational [36] studies. D162 is inferred from these studies to play a critical role in properly positioning two lysines (K8, K217) that stabilize the negative charge of ATP. The minimal displacements of D162 and K8 in these global modes, and the intrinsic tendency of K217 to move toward D162, are presumably dynamic requirements to optimally perform their catalytic roles (note that D162 is located close to the hinge site of GNM mode 2, as pointed out above, and thus its mobility is rather constrained). This rigidity is confirmed by the striking similarity in the orientation of these residues in different bound states of the enzyme [33] that characterize the entire catalytic process. The rearrangements of catalytic residues may be necessary to optimally orient, or pre-organize, the ligands to catalyze the chemical reaction [13],[14],[38]. In this case, some additional changes appear to occur in the bound form, such as the change in the side chain conformation of K217, which exchanges a salt bridge between residues E181 and D162. These rearrangements would presumably take place upon ligand binding, since E181 interacts with ATP via hydrogen bonds.

In relation to the NAG binding process, mutants on the NAG binding site revealed that N158 and R66 are key residues that underlie the affinity of *Ec*NAGK for NAG [36]. By examining the NAG binding mode (5^th^ ANM mode, see Figure S3), R66 was found to be far more flexible than N158. This suggests that R66 may play a role in the recognition of the ligand, whereas the less exposed residue N158 might subsequently aid to fix the position of NAG at the active site. Furthermore, upon NAG binding, the size of the hydrophobic pocket (L65, R66, V122 and N160) that hosts the methyl group of NAG is reduced upon correlated movements between R66 and L65 toward the closed form. Binding of R66 and N158 to NAG, thus, apparently guides L65 toward the methyl group of the substrate. The correlated movements of L65 together with the rigidity of V122 and N160 fix the size of the hydrophobic pocket, which has been observed to be unable to bind glutamate derivatives with larger N-acyl groups [32],[39] (Figure S3).

### Communication properties

Using the Markov model described in the *Methods*, we computed the *hitting times H_ji_*. *H_ji_* provides a measure of the average path length over all possible combinations of edges, required to send information to a given node *j*, or ‘hit’ residue *j*, starting from node *i*. The hitting times for all pairs of residues were evaluated for three different cases: open form (NAGK(O)), closed from without ligands (NAGK(C)) and closed form with ligands (NAGK(C)+ligands). Toward gaining an understanding of the communication propensity of individual residues, results have been consolidated, by calculating the mean hitting time, <*H_i_*> = (1/N) Σ_j_ *H_ji_*, for each residue *i*.

**Figure 6:**
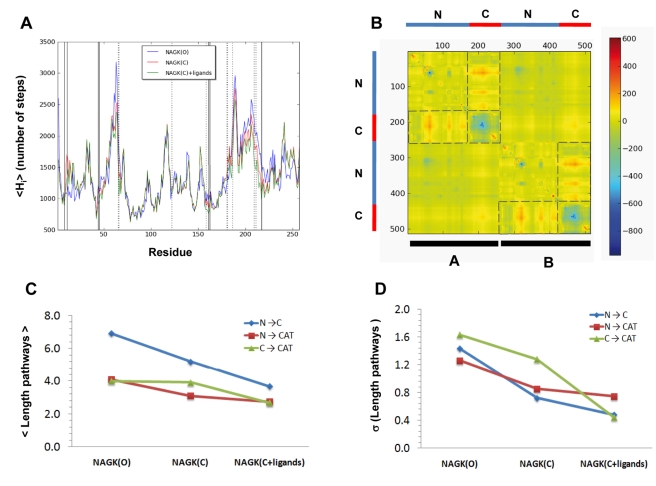
Communication properties of NAGK. (A) Mean hitting time profile for the open and closed (with and without ligands) forms of NAGK. Vertical lines indicate the positions of catalytic (solid line) and ligand-binding residues (dotted line). Note that catalytic residue tend to occupy minima positions, indicative of their efficient communication properties. (B) Difference map between the contribution to hitting times from cross-correlations (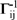) (equation (8) in *Methods*) of the open and ligand-bound closed forms. Dashed lines set the boundaries of N- and C- domains and also enclose those pairs of domains that undergo the largest changes in the contribution from cross-correlations upon ligand binding. (C) Mean path lengths for linking different parts of the protein: N- and C-domains (blue), N-domain and catalytic site (red), and C-domain and catalytic site (green). (D) Standard deviation in the mean paths displayed in panel (C).

Figure 6A displays the mean hitting times of all residues in the three cases. The main contribution to *H_ji_* arises from the MSF of the target residue itself (via the term [**Γ** ^−1^]*_jj_* in equation (8), which in turn is proportional to <(Δ*R_j_*)^2^> - see equation (3)). As a result, the average hitting time profile shares some characteristics with the MSF profile shown in Figure 2B. The minima correspond to the most efficient receivers; these exhibit minimal fluctuations in their positions. It is worth noting that catalytic residues are among those with the lowest hitting times. These results suggests that the structural position and contact topology of the active site have been evolutionary designed to effectively receive signals from the binding sites and other parts of the protein so as to optimize the catalytic activity of the enzyme. On the other hand, the ligand-binding residues exhibit a broader variety of hitting times.

A closer comparison of the results obtained for the three structures revealed an interesting feature upon examination of the average hitting times between different substructures. The results in Figure 6C display the average path lengths evaluated for the communication between such particular domains in each structure: the average path lengths over all residue pairs belonging to the respective C-terminal and N-terminal domains (blue curves), those over all the N-terminal domain and catalytic site residues (red), and those over the C-terminal domain and catalytic site residues (green). These results clearly demonstrate that the closure of the structure enhances the communication of residues (decreases the average hitting times or path lengths) and upon ligand binding the communication shows a further improvement. Panel (D) demonstrates that not only the average path lengths, but the variance in the path lengths decrease upon domain closure, and ligand binding. In all cases, the N- and C-domains exhibit average path lengths longer than those connecting either domain to the catalytic site. This is a natural consequence of the location of catalytic residues - at the inter-domain region, where the phosphoryl transfer takes place.

**Figure 7:**
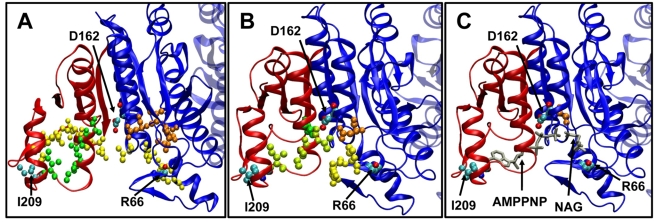
Communication pathways between catalytic site and ligand-binding residues in *Ec*NAGK. The communication pathways are represented by the network of residues (each atom is shown as a dot) along the shortest paths of communication between the following residue pairs: R66-L209 (yellow), R66-D162 (orange) and L209-D162 (green). These three cases are representative of the communication between a NAG-binding residue (R66) on the N-domain, a catalytic site residue (D162) and an AMPPNP binding residue on the C-domain (L209). These residues at the endpoints of the pathways are colored by atom name. N- and C- domains are colored blue and red, respectively. These pathways are shown for the three states considered: (A) Open state. (B) Closed state. The pathways R66-L209 and L209-D162 have three residues in common (colored in light green). (C) Closed state with ligands AMPPNP and NAG. The ligands are shown with gray sticks for a better visualization and participate in all three pathways considered in the figure. Note that the ligands directly establish the communication between the pairs of residues considered.

Figure 7 panels (A) and (B) illustrates the three types of communication pathways in the open and closed states. Three residues have been selected as endpoints representative of the N-terminal domain NAG-binding site (R66), C-terminal domain nucleotide-binding site (L209) and the catalytic site at the inter-domain interface (D162), and the residues along the shortest paths evaluated using the Dijkstra's algorithm (see *Methods*) are shown by different dots in each case (see caption). We note in panel (C) that the ligand in the closed+liganded structure effectively spans the optimal communication pathway.

The enhancement of communication observed in panels (C) and (D) of Figure 6 is a consequence of the rigidity imparted by the closure of the structure and by ligand binding. The structure obviously becomes more cohesive in the closed conformation and consequently the couplings between residue fluctuations are increased, or the fluctuations in inter-residue distances are reduced. As summarized in the methods and derived in detail in our previous work [40], the commute times τ_ij_ between residue pairs directly scale with the fluctuations in the corresponding inter-residue distances (see equation (9)). Restrictions in inter-residue distance fluctuations acquired upon closure of the structure thus necessarily induce an enhancement in communication. From a catalytic point of view, the closure of the structure upon substrate binding is presumably an efficient way to optimize signal transduction and facilitate the catalytic process. Panel (B) in Figure 6 displays the changes in the contribution to hitting times from cross-correlations (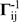) evaluated using equation (8) (see *Methods*) twice, for the open and closed+liganded states, and taking their difference. It is observed that upon domain closure and ligand binding the contribution from cross-correlations within the C-domain decrease (blue points in the panel), whereas those between the N- and C-domains within each subunit increase (red points in the panel). The resultant shorter and more homogeneous communication pathways suggest that ligands tend to centralize the communication between the C- and N- domains. Therefore, the transmission of conformational signals between the flexible domains and the more rigid catalytic residues takes place across the substrates. This might indicate a way to cooperatively optimize substrate binding (or product release) or even couple the intrinsic enzyme dynamics to the catalysis of the chemical reaction.

### Comparison of EcNAGK dynamics with other members of the AAK family

**Figure 8:**
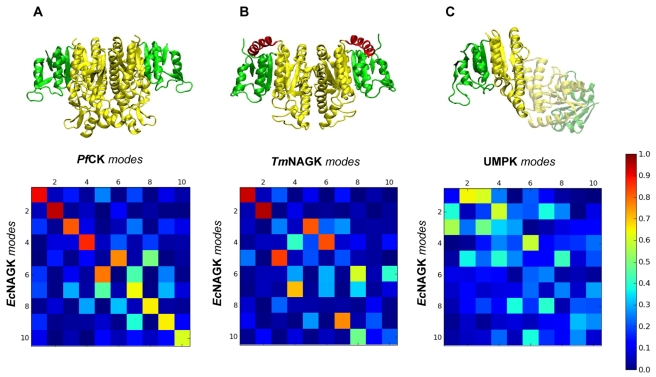
Conservation of the lowest frequency modes of motion among the AAK family members. Correlation cosines between the ten slowest modes of *Ec*NAGK and those of three other members of the AAK family. (A) Dimeric *Pf*CK. (B) *Ec*NAGK-like dimer of *Tm*NAGK. (C) Monomer of *Pf*UMPK. Ribbon diagrams represent the structure of the corresponding dimers of the AAK family that are compared with *Ec*NAGK. The C- and N-domains are colored green and yellow. In panel B, the N-terminal helix (red) is a key element for the hexamerization of *Tm*NAGK. In panel C, one of the monomers is shadowed to highlight that it has been considered as the coupling environment of the monomer that is being compared with a monomer of *Ec*NAGK.

It is of interest to determine if the dynamical features observed for *Ec*NAGK are shared by other members of the AAK family. Various approaches can be adopted to this aim [19],[21],[41]. Here, we focus on global dynamics and compare the ANM modes predicted for *Ec*NAGK with those predicted for each AAK member. First, each pair of enzymes is structurally aligned using the DALI server [42]. Second, we define our ‘subsystem’ as the aligned portions of the families members and constructed the Hessian submatrices for these portions, and the remaining chain segments are considered as environment, similar to the approach adopted by Zheng & Brooks [43], described in *Methods*). Third, NMA is performed using equation (11) for the Hessian of the examined system. The correlation cosines between the collective modes evaluated for each subsystem and those calculated for the *Ec*NAGK dimer are presented in Figure 8. Results are shown for the top-ranking 10 modes, calculated for the corresponding dimers of three different members of the AAK family (structures shown in Figure 8): Carbamate kinase from *Pyrococcus furiosus* (*Pf*CK), NAGK from *Thermotoga Maritima* (*Tm*NAGK) and UMPK from *Pyrococcus furiosus* (*Pf*UMPK). Further information on the structural and dynamical pairwise comparisons is provided in Table 1.

**Table 1:** Structural and dynamical similarities among the AAK family members.

Pairwise comparison	Slow modes overlap (a) (%)	RMSD (Å)	Structurally aligned positions(b) (%)	Sequence identity at aligned positions (%)
*Ec*NAGK/*Pf*CK	88	3.9	91	20
*Ec*NAGK/*Tm*NAGK	81	3.3	99	32
*Ec*NAGK/*Pf*UMPK	70	3.3	77	16

(a) <CO(m)>, for m = 10, averaged over the first 10 modes.

(b) Residue pairs of proteins A and B are aligned by minimizing the deviations of intramolecular α-carbon distances (r_ij_^A^ and r_ij_^B^) relative to their arithmetic mean. A threshold of similarity is set to 20%.

#### *EcNAGK vs. Pf*CK (Figure 8A)

The crystallographic structure of *PfCK* (PDB code 1E19) represents the open form of the enzyme [44]. Remarkably high correlations are obtained between the slow modes accessible to the two enzymes as may be seen in Figure 8A.

#### *EcNAGK vs. TmNAGK* (Figure 8B)

*Tm*NAGK is a hexamer that can be regarded as a trimer of *Ec*NAGK-like dimers (PDB code 2BTY). Thus, we compared the slow modes of *Ec*NAGK with those sampled by the *Ec*NAGK-like dimer from *Tm*NAGK. The five lowest modes of *Ec*NAGK are almost identically observed in *Tm*NAGK, except for a change in the order (or relative frequencies) of the modes.

#### *EcNAGK vs. PfUMPK* (Figure 8C)

UMPK is also a trimer of dimers [45] (PDB code 2BRI), but the monomeric subunits within these dimers are not arranged in the same manner as *Ec*NAGK or *Tm*NAGK. Thus, the comparison has been made between the dynamics of the monomeric subunits of *Ec*NAGK and *Pf*UMPK, including as environment, *via* equation (11), the remaining residues of the respective dimers. The mode-mode correspondences are not as clear as in the previous cases, but there is still some discernible correlation. The weaker correspondence can be attributed to the fact that the percentage of aligned residues (77%) is lower than that of the previous two cases (above 90%) (see Table 1).

It is worth pointing out that the modes of motion of the dimeric scaffold of the hexameric enzymes (*Tm*NAGK and *Pf*UMPK) may well be affected by the interfaces with the rest of subunits. The analysis of oligomerization effects on the dynamics of these proteins, however, is beyond the scope of this article and will be published elsewhere. The three cases studied here illustrate how the slow conformational dynamics of the *Ec*NAGK dimer is preserved to a large extent among the members of the AAK family. It is worth emphasizing that some of the modes are remarkably well conserved. In accordance with other studies [19], the lowest frequency modes prove here to be robust to sequence and structural variations within a given protein family; and the shared dynamics may be viewed as a dynamic fingerprint of the AAK family.

### Conclusions

The present study focused on the *Ec*NAGK dimer in order to provide new insights into the competition between intrinsic *vs* induced dynamics in controlling enzymatic activity, assessing which residues play a key role in mediating the collective motions, or which conformational mechanisms are shared among members of the AAK family. The most probable modes of motion encoded by the structure have been determined using the available structural data for *Ec*NAGK dimer, which is used as a prototype, as well as other members of the family. The present study illustrates that this family, not only has important sequence and structure similarities, but also shares relevant dynamical features (Figure 8).

The results in Figure 4 demonstrate that the conformational change observed between the open and closed forms of *Ec*NAGK are essentially accomplished by movements along a small subset of modes (among the complete set of 1542 modes accessible to the enzyme); these are the modes predicted by the GNM to be the softest, i.e., they incur the least ascent in energy for a given size of motion. This shows that the ‘easiest’ movements intrinsically favoured by the enzyme structure are actually those underlying its ligand binding mechanism, suggesting an evolutionary optimization of structure to favour functional dynamics.

Closer examination of the changes on a local scale, on the other hand, shows that the changes induced at residue level, may have the right directions to facilitate the interactions with the substrate, but are not sufficiently large and specific enough to explain the re-positioning of amino acids near active sites. The ATP-bound conformation appears, for example, to result from the intrinsic dynamics of the protein combined with local rearrangements induced by the ligand to optimize its interactions in the complex (Figure 5).

The low frequency modes shared among the examined family members are shown here to enable access to the active site, by opening/closing to the environment the cleft where catalytic residues reside. Notably, these movements have minimal effects on the organization of catalytic residues, which are located near the hinge center that allows for the relative movements of the N- and C-domains in each subunit. The restricted mobility of catalytic residues is consistent with previous observations where catalytic sites have been pointed out to be highly constrained in the global modes and occupy key positions (near or coinciding with global hinges) in the structure. The collective modes do not therefore induce distinctive rearrangements at the catalytic residues. However, they appear to modulate their communication to the environment, i.e. they provide exposure to solvent, and/or a loosening or tightening of the packing density in the neighbourhood which apparently plays a role in controlling the propagation of structural or energetic perturbations to/from the active site.

Perhaps the most striking results concern the communication properties and the role of domain movements and ligand binding in enhancing allosteric effects. The decrease in the mean values and dispersion of the hitting times and the communication path lengths upon ligand-binding (Figure 6) suggests that the ligands optimize the coupling of domain movements that are already characteristic of the intrinsic protein dynamics. Indeed, the structure may have been evolutionary selected to bind the substrates in an optimal position to maximize the allosteric couplings.

## Methods

The protein structure is represented as an ENM. The coordinates in the native structure are assumed to define the equilibrium positions of network nodes/residues. Pairs of nodes within a cutoff distance are coupled by elastic springs. Although the inter-residue interactions are non-specific, the collective dynamics of the protein in the low frequency regime is primarily and robustly determined by the overall fold [17],[46],[47], which permits us to explore the cooperative motions of NAGK using ENMs. Different ENMs have been developed [23], [48]–[51], and Phillips and co-workers [52] demonstrated that these provide a consistent description of the lowest-frequency modes. Here we adopt the GNM [51],[53] and the ANM [23].

### Gaussian Network Model (GNM)

The GNM potential depends on the vectorial distance between each pair of nodes as
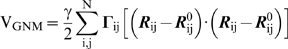
(1)where *N* is the total number of residues, γ the uniform force constant for all springs in the network, **Γ** *_ij_* is the *ij^th^* element of the *N*×*N* Kirchhoff matrix **Γ** that defines the connectivity of the network, equal to −1 if residues *i* and *j* are within a cutoff distance R_c_, zero otherwise, **R**_ij_ and **R**_ij_^0^ are the instantaneous and equilibrium distance vectors between residues *i* and *j*, residue positions being identified by those of their α-carbons in the PDB files. The GNM approach allows for decomposing the dynamics of the protein into a set of normal modes of motion upon eigenvalue decomposition of **Γ**. The contribution of the *k* ^th^ mode to the MSF of residue *i* is expressed as
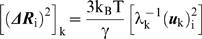
(2)where λ_k_ and ***u_k_*** are the *k* ^th^ eigenvalue and eigenvector of **Γ**, respectively; (***u*** *_k_*)_i_ designates the mobility of residue *i* along the *k* ^th^ mode. The low-frequency modes usually have the highest degree of collectivity, and they make the largest contribution to the observed MSFs
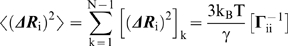
(3)where the summation is performed over all non-zero modes and [**Γ** ^−1^]*_ii_* designates the *i^th^* diagonal element of the inverse of **Γ**. Therefore, the lowest frequency modes usually provide insights into the cooperative motions involved in biological function [22].

GNM provides information on the relative sizes of residue motions in different modes (equation (2)), the MSFs of individual residues (equation (3)), or their cross-correlations
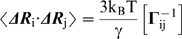
(4)(obtained by rewriting equation (3) for pairs of residues *i* and *j*), as but not on their directionality; the fluctuations are implicitly assumed to be isotropic. The 3D characterization of the normal modes is provided by the ANM.

### Anisotropic Network Model (ANM)

The ANM potential is a function of the scalar distance between the interacting pair of nodes and is given by [23]

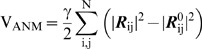
(5)Both GNM and ANM penalize inter-residue distance changes, but GNM also takes into account the orientational change of the inter-residue vector, which leads to better agreement with experimental *B*-factors [54]. NMA is carried out using the 3*N*×3*N* Hessian matrix **H** derived from the ANM potential. The diagonalization of **H** yields 3*N*-6 non-zero modes (as opposed to *N*-1 in the GNM). A given ANM mode (eigenvector) thus contains information on the x-,y- and z-components of the motion undergone by each residue, thus describing the spatial directionalities of the collective motions.

### Generation of large-amplitude conformational changes

ANM modes can be used to generate alternative conformations sampled along most easily accessible (lowest frequency) mode directions. Due to the harmonic character of the potential, two sets of conformers are obtained for a given mode *k*:
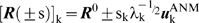
(6)where λ_k_ and 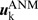 are the eigenvalue and eigenvector for mode *k* respectively, ***R^0^*** is the 3*N*-dimensional vector representing the initial coordinates and *s_k_* is a parameter that scales the amplitude of the deformation induced by mode *k*. No sidechain atomic coordinates are included in the ANM calculations. An all-atom model for the deformed structure is generated by displacing the backbone and side chain atoms of each residue along the mode component of the corresponding C^α^-atom and subsequent energy minimization. Such energy minimization performed with Gromacs [55] was verified to involve negligible conformational change in the backbone.

### Comparison of experimental conformational changes with ANM modes

The degree of similarity between a conformational change *Δ* ***r*** observed by crystallography and the theoretically predicted direction of the *k^th^* mode can be quantified with the correlation cosine, *cos*(*Δ* ***r***·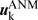). Here *Δ* ***r*** refers to the 3N-dimensional difference vector between the α-carbon coordinates of the open form and closed form of NAGK, for example, after optimal superimposition of the two structures to eliminate the external degrees of freedom. The cumulative overlap between the experimentally observed deformation *Δ* ***r*** and that accounted for by a subset of *m* modes (*m *< 3*N*-6) is given by a summation of squared correlation cosines as
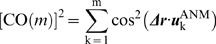
(7)The summation of the squared cosines over all 3*N*-6 nonzero modes is identically equal to unity as the eigenvectors form a complete orthonormal basis set in the 3*N*-6 dimensional space of internal conformational changes. In the absence of correlation between ***Δr*** and 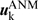, the average correlation cosine squared contributed by mode *k* will thus be 1/(3*N*-6). Note the strong departure from this random behaviour in Figure 4.

### Communication propensities. Hitting times and relation to collective dynamics

Inter-residue communication has been suggested as an essential mechanism in the allosteric regulation of protein function and enzymatic catalysis [56], and explored in diverse computational studies [57]–[59]. A network-based Markov model has been recently developed [40],[60], which reconciles the NMA-based predictions on allosteric changes in conformations (global modes) with the shortest path(s) analyses based on graph theoretical methods [28]. We use this method to identify communication paths. The interactions between residue pairs are defined therein by the affinity matrix **A**. The elements of this matrix are defined as [60] a_ij_ = N_ij_/(N_i_ N_j_)^½^ where N_ij_ is the number of atom-atom contacts between residues *i* and *j*, based on a threshold distance of 4 Å, and N_i_,N_j_ are the number of heavy atoms of both residues. **A** is related to the Kirchhoff matrix 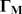 (same as GNM 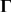, except for the adoption of affinities, instead of γ, for the weights of the edges) as 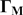 = **D**−**A**, where **D** is the diagonal matrix of elements 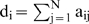. In the simplified case where a_ij_ = 1 for all R_ij_<R_c_, 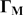 reduces to the GNM 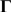. The network communication is controlled by the Markov transition matrix **M** = {*m_ij_*}, where *m_ij_* = *a_ij_*/*d_j_* represents the conditional probability of transmitting a signal from residue *j* to residue *i* in one time step [60]. We define −log(*m_ij_*) as the ‘distance’ between two residues, in terms of communication, and the maximum-likelihood paths associated with each residue pair are evaluated using the Dijkstra's algorithm [60]. This permits us to evaluate a basic communication property: hitting time **H**_ji_ as the average path length for the passage of signals from node *i* to node *j* [40]. **H**_ji_ can be expressed in terms of the elements of 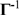 (or 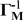) as [40]

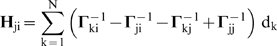
(8)Given that the elements of **Γ** ^−1^ scale with the MSFs of residues (diagonal elements) or the cross-correlations between residue fluctuations (off-diagonal elements), the above equation establishes the link between the signal transduction properties of the protein and its collective dynamics [40]. Note that the commute time **τ**_ij_ = **H**_ij_ + **H**_ji_ assumes an even simpler form, using equation (8) twice, i.e.,
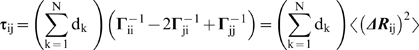
(9)This equation simply states that the communication between two residues takes longer if their inter-residue distances have higher fluctuations [40]. The inter-residue distances, in turn, are readily evaluated from the difference <(Δ***R*** *_ij_*)^2^> = <(Δ***R*** *_i_*)^2^> + <(Δ***R*** *_j_*)^2^> −2 <(Δ***R*** *_i_* • Δ***R*** *_j_*>, where the respective terms are evaluated using the equations (3) and (4).

### NMA of a subsystem coupled to a dynamic environment

If the dynamics of a part of the protein (subsystem, S) in the presence of an environment (E) is of interest, a useful approach is to partition the Hessian into four submatrices [43]:
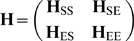
(10)where **H**_SS_ is the matrix referring to the subsystem, **H**_EE_ to that associated with the interactions within the environment and **H**_SE_ (and **H**_ES_) to those coupling the subsystem to the environment. An effective Hessian for the subsystem 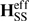 can be constructed from these elements as
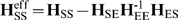
(11)The NMA of 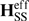 effectively describes the collective dynamics of the subsystem in the presence of coupling to the environment. This approach proved useful in determining the allosteric potential of residues [61] or the location of transition states of chemical reactions by defining a reduced potential energy surface [62].

The above method has been used for evaluating the overlap between the collective modes of different family members in different environments. The cumulative overlap (expressed in terms of percentage in Table 1) between subsets of modes is evaluated using equation (7) where a double summation over the particular subsets of modes of interest, e.g. top ranking 10 modes of the two systems.

All figures depicting molecular structures have been generated with the VMD visualization software [63].

## Supporting Information

Figure S1 Representation of the movement undergone by EcNAGK in ANM modes 1,3 and 5. Ribbon diagrams represent deformed conformations generated using Eq 5. Different perspectives of the enzyme (see rotation of the reference axes) have been displayed to highlight the main deformation of each mode: front view (mode 1), lateral view (mode 3) and bottom view (mode 5). C and N domains are colored in red and blue respectively.
(3.62 MB TIF)

Figure S2 Movement of active site residues between open and closed conformers along the 3rd ANM mode accessible to the open form. The position of these residues in different conformations is shown: open conformation (yellow), intermediate positions (green and blue) and closed conformation (atom-colored). (A) Color-coded ribbon diagram for motions along the 3rd mode (generated with the ANM web server[1] and Pymol[2]). (B) Movement of catalytic residues with respect to the ATP analogue. (C) Movement of ATP binding residues with respect to the nucleotide. (D) Movement of NAG binding residues with respect to NAG. 1. Eyal E, Yang LW, Bahar I (2006) Anisotropic network model: systematic evaluation and a new web interface. Bioinformatics 22: 2619–2627. 2. DeLano WL (2002) The PyMOL Molecular Graphics System. San Carlos, CA: DeLano Scientific.
(2.06 MB TIF)

Figure S3 Movement of NAG binding residues between open and closed conformers along the 5th ANM mode accessible to the open form. (A) The position of these residues in different conformations is shown: open conformation (yellow), intermediate positions (green and blue) and closed conformation (atom-colored). (B) Schematic representation of the conformational change of the hydrophobic pocket at the NAG binding site along the 5th ANM mode. Red dots show the interaction sites between NAG and residues R66 and 158. Residues R66 and L65 move concertedly toward the interaction site of N158, which fixes the size of the hydrophobic pocket.
(0.64 MB TIF)
