# Supplementary figures and images for: On the Conservation of the Slow Conformational Dynamics within the Amino Acid Kinase Family: NAGK the Paradigm
Source: PLoS Comput Biol. 2010 Apr 8;6(4):e1000738. doi: 10.1371/journal.pcbi.1000738 (PMC2851564; doi:10.1371/journal.pcbi.1000738)

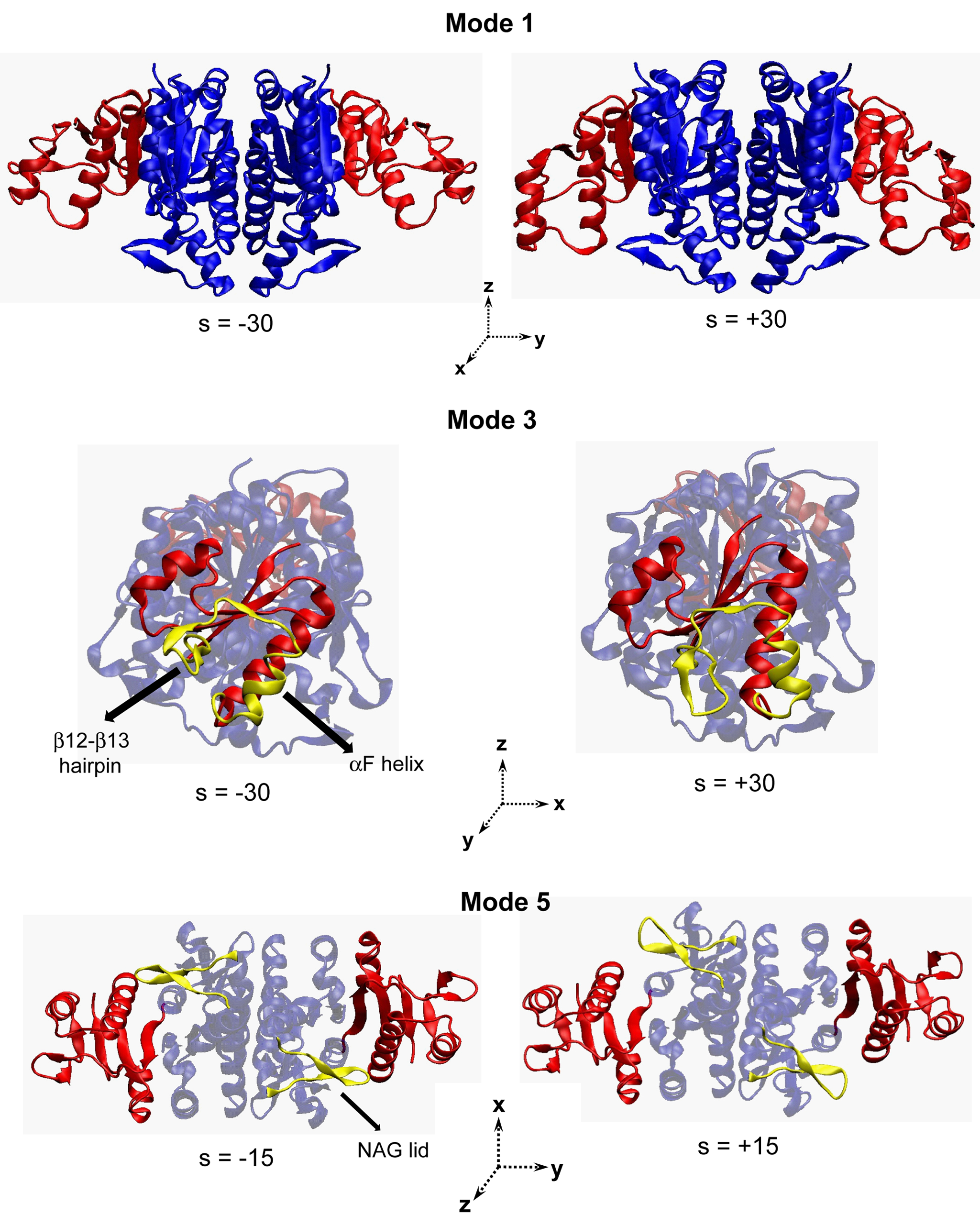

Supplement: Figure S1 — Representation of the movement undergone by EcNAGK in ANM modes 1,3 and 5. Ribbon diagrams represent deformed conformations generated using Eq 5. Different perspectives of the enzyme (see rotation of the reference axes) have been displayed to highlight the main deformation of each mode: front view (mode 1), lateral view (mode 3) and bottom view (mode 5). C and N domains are colored in red and blue respectively. (3.62 MB TIF) [file pcbi.1000738.s001.tif]

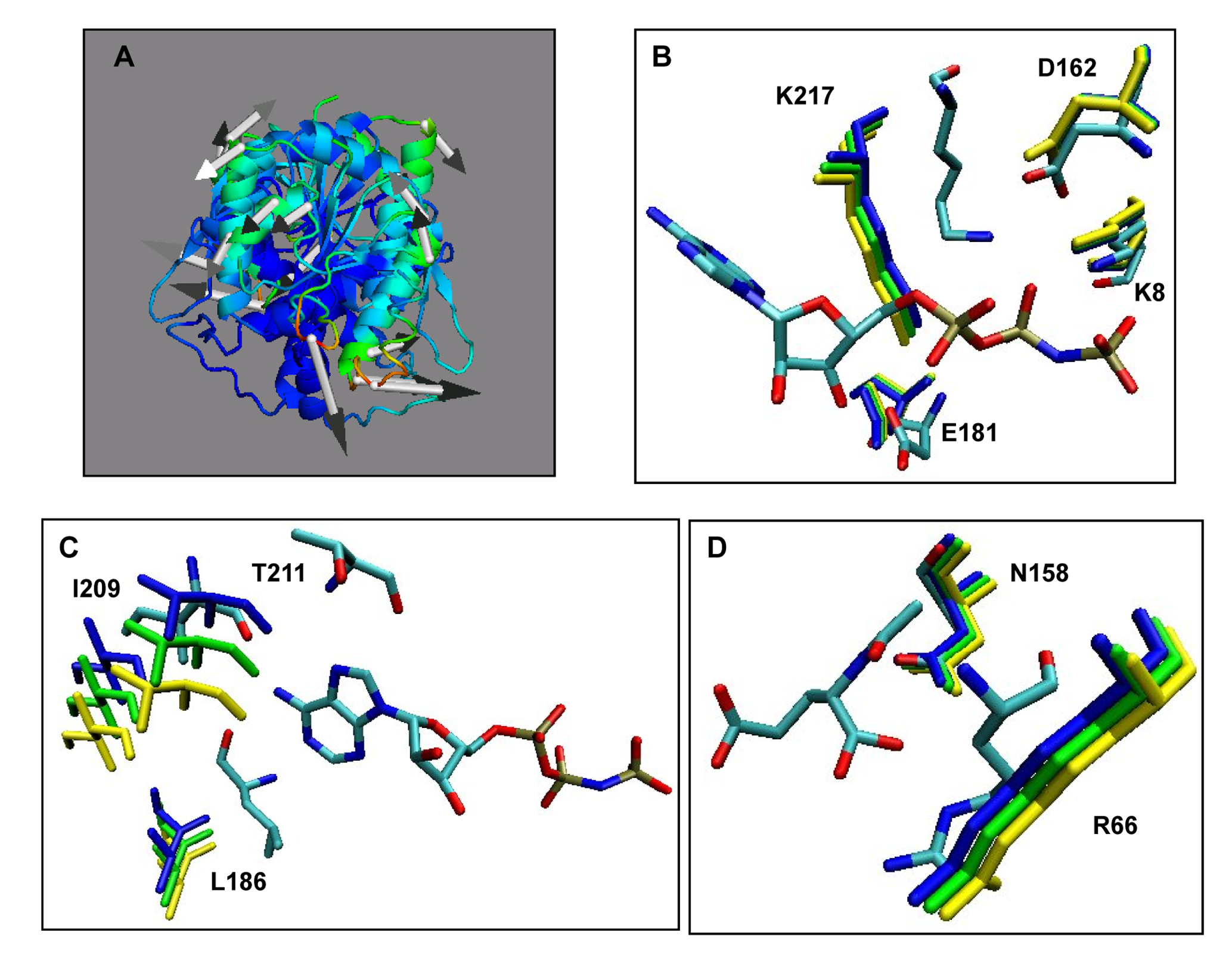

Supplement: Figure S2 — Movement of active site residues between open and closed conformers along the 3rd ANM mode accessible to the open form. The position of these residues in different conformations is shown: open conformation (yellow), intermediate positions (green and blue) and closed conformation (atom-colored). (A) Color-coded ribbon diagram for motions along the 3rd mode (generated with the ANM web server[1] and Pymol[2]). (B) Movement of catalytic residues with respect to the ATP analogue. (C) Movement of ATP binding residues with respect to the nucleotide. (D) Movement of NAG binding residues with respect to NAG. 1. Eyal E, Yang LW, Bahar I (2006) Anisotropic network model: systematic evaluation and a new web interface. Bioinformatics 22: 2619–2627. 2. DeLano WL (2002) The PyMOL Molecular Graphics System. San Carlos, CA: DeLano Scientific. (2.06 MB TIF) [file pcbi.1000738.s002.tif]

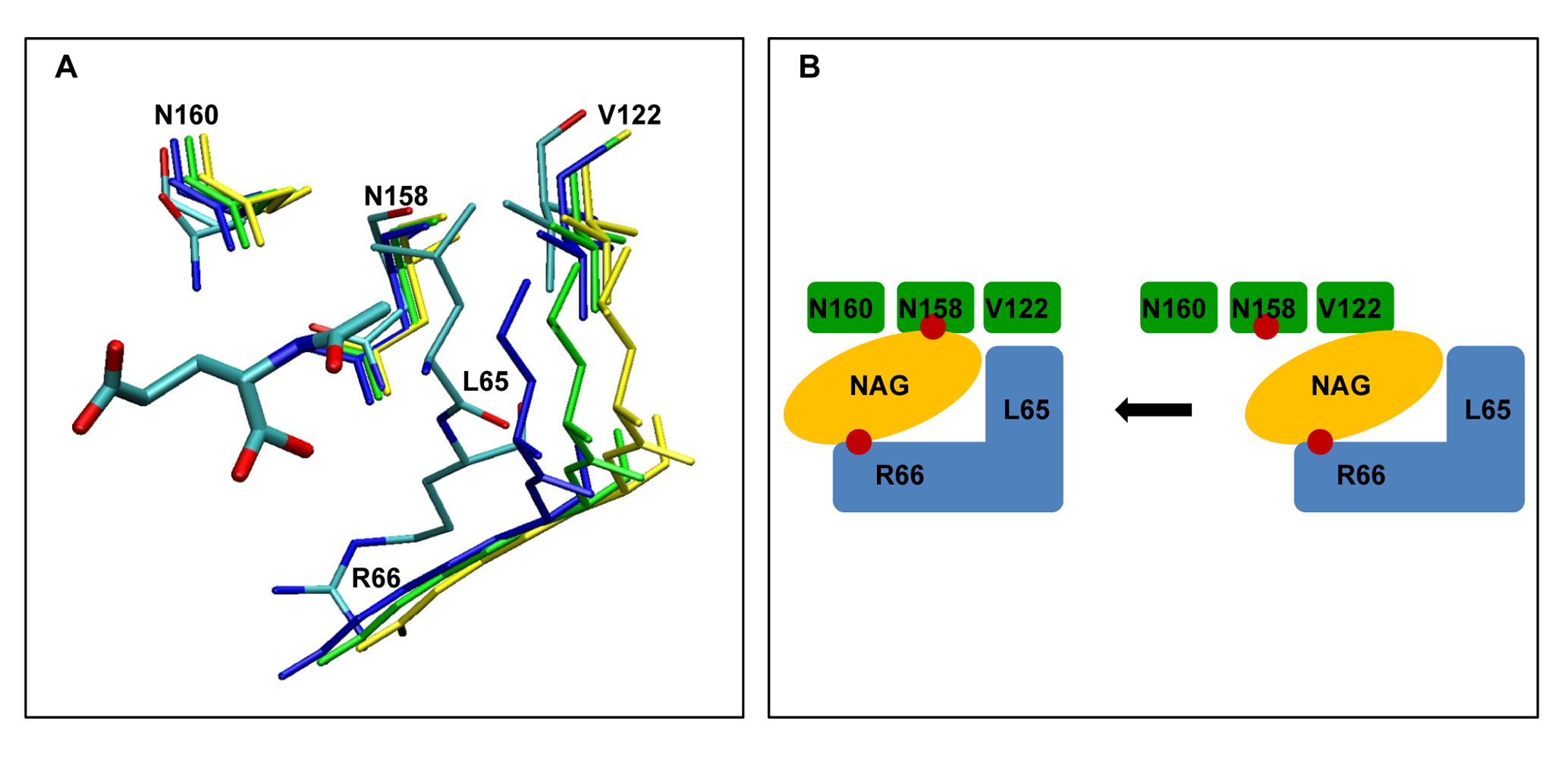

Supplement: Figure S3 — Movement of NAG binding residues between open and closed conformers along the 5th ANM mode accessible to the open form. (A) The position of these residues in different conformations is shown: open conformation (yellow), intermediate positions (green and blue) and closed conformation (atom-colored). (B) Schematic representation of the conformational change of the hydrophobic pocket at the NAG binding site along the 5th ANM mode. Red dots show the interaction sites between NAG and residues R66 and 158. Residues R66 and L65 move concertedly toward the interaction site of N158, which fixes the size of the hydrophobic pocket. (0.64 MB TIF) [file pcbi.1000738.s003.tif]
